# Coptisine-induced inhibition of *Helicobacter pylori*: elucidation of specific mechanisms by probing urease active site and its maturation process

**DOI:** 10.1080/14756366.2018.1501044

**Published:** 2018-09-07

**Authors:** Cailan Li, Ping Huang, Kambo Wong, Yifei Xu, Lihua Tan, Hanbin Chen, Qiang Lu, Chaodan Luo, Chunlai Tam, Lixiang Zhu, Ziren Su, Jianhui Xie

**Affiliations:** a Guangdong Provincial Key Laboratory of New Drug Development and Research of Chinese Medicine, Mathematical Engineering Academy of Chinese Medicine, Guangzhou University of Chinese Medicine, Guangzhou, P. R. China;; b School of Pharmaceutical Sciences, Guangzhou University of Chinese Medicine, Guangzhou, P. R. China;; c School of Life Sciences, Center for Protein Science and Crystallography, The Chinese University of Hong Kong, P. R. China;; d The First Affiliated Hospital of Chinese Medicine, Guangzhou University of Chinese Medicine, Guangzhou, P. R. China;; e Key Laboratory of Chinese Medicinal Resource from Lingnan, Ministry of Education and Research Center of Chinese Herbal Resource Science and Engineering, Guangzhou University of Chinese Medicine, Guangzhou, P. R. China;; f Guangdong Provincial Key Laboratory of Clinical Research on Traditional Chinese Medicine Syndrome, The Second Affiliated Hospital, Guangzhou University of Chinese Medicine, Guangzhou, P. R. China

**Keywords:** coptisine, *Helicobacter pylori* urease, sulfhydryl group, nickel ion, UreG

## Abstract

In this study, we examined the anti-*Helicobactor pylori* effects of the main protoberberine-type alkaloids in Rhizoma Coptidis. Coptisine exerted varying antibacterial and bactericidal effects against three standard *H. pylori* strains and eleven clinical isolates, including four drug-resistant strains, with minimum inhibitory concentrations ranging from 25 to 50 μg/mL and minimal bactericidal concentrations ranging from 37.5 to 125 μg/mL. Coptisine’s anti-*H. pylori* effects derived from specific inhibition of urease *in vivo*. *In vitro*, coptisine inactivated urease in a concentration-dependent manner through slow-binding inhibition and involved binding to the urease active site sulfhydryl group. Coptisine inhibition of *H. pylori* urease (HPU) was mixed type, while inhibition of jack bean urease was non-competitive. Importantly, coptisine also inhibited HPU by binding to its nickel metallocentre. Besides, coptisine interfered with urease maturation by inhibiting activity of prototypical urease accessory protein UreG and formation of UreG dimers and by promoting dissociation of nickel from UreG dimers. These findings demonstrate that coptisine inhibits urease activity by targeting its active site and inhibiting its maturation, thereby effectively inhibiting *H. pylori*. Coptisine may thus be an effective anti-*H. pylori* agent.

## Introduction

*Helicobacter pylori*, a gram-negative, spiral-shaped, microaerophilic bacillus, is the major pathogen underlying multiple digestive system diseases, including gastritis, ulcers, gastric carcinoma, and related gastroduodenal disorders^1–3^. It affects approximately 50% of the population worldwide^4^ and is classified as a class I carcinogen by the World Health Organization^5^. The first-line therapy for *H. pylori* eradication usually involves combination of a proton-pump inhibitor (e.g. lansoprazole) and two antibiotics (e.g. clarithromycin and metronidazole or amoxicillin). Although it is somewhat efficacious^6^^,^^7^, there are substantial challenges associated with this regime, including poor patient compliance, antibiotic resistance, possible recurrence of infection, and the development of other gastrointestinal diseases due to disturbances of the gut microbiome^8^^,^^9^. Identification of novel drug candidates, especially those with natural origins, is therefore a topic of ongoing research^10–12^.

Urease (EC 3.5.1.5; urea amidohydrolase), which is critical to the colonisation and virulence of *H. pylori*, is one of the most important targets for the treatment of *H. pylori* infection^13^. Urease is a nickel-dependent metalloenzyme that catalyses the hydrolysis of urea to ammonia, thus neutralising gastric acid and creating an alkaline local environment required for the survival of *H. pylori*^14^. Urease therefore plays a crucial role in the pathogenesis of gastric and peptic ulcers, gastric cancer, and gastric adenocarcinoma^15^, and strategies that inhibit urease are essential for the treatment of *H. pylori* infection.

The binuclear nickel ions (Ni^2+^) in the active site and sulfhydryl groups situated in the mobile flap are essential for urease’s catalytic activity^16^^,^^17^. Indeed, hydroxamic acids, phosphoramidates, and urea derivatives interacting with the metallocentre^18^^–^^20^, and quinones and polyphenols binding to the sulfhydryl groups of the urease active site^21^^,^^22^, constitute the current major structural classes of urease inhibitors. Moreover, it has been reported that GTP hydrolysis and the formation of a pre-activation complex consisting of apourease and four urease accessory proteins, UreE, UreF, UreG, and UreH (an ortholog of UreD found in other species), are required for carbamylation of Lys219 and insertion of two nickel ions into its active site, which contribute to the maturation of urease^23–25^. UreG, a SIMIBI (signal recognition, MinD, and BioD) class GTPase, is recruited by the UreF–UreH complex and is responsible for coupling GTP hydrolysis and regulating nickel delivery during the process of urease activation^26^. The biological functions of UreG are frequently regulated by dimerisation^27^. Agents targeting the essential catalytic mechanism of urease could result in its inactivation and therefore provide an invaluable contribution to the treatment of *H. pylori* infection^13^.

Rhizoma Coptidis (the rhizoma of *Coptis chinensis* Franch., called Huanglian in Chinese), which is officially listed in the *Chinese Pharmacopoeia*, is one of the most commonly used traditional Chinese medicines for the treatment of *H. pylori*-related gastrointestinal diseases. Protoberberine alkaloids, such as berberine, coptisine, palmatine, epiberberine, and jatrorrhizine, which share a common core structure, are considered the major active components of Rhizoma Coptidis. Recent research on Coptidis alkaloids has focussed primarily on berberine, the most abundant alkaloid in Rhizoma Coptidis. Reports indicate that Rhizoma Coptidis extract usually has stronger anti-*H. pylori*^28^, anti-enzyme^29^, gastroprotective^30^, and anti-cancer effects^31^ than pure berberine, indicating that components other than berberine also play an important role in the extract’s biological activities.

**Figure 1.**
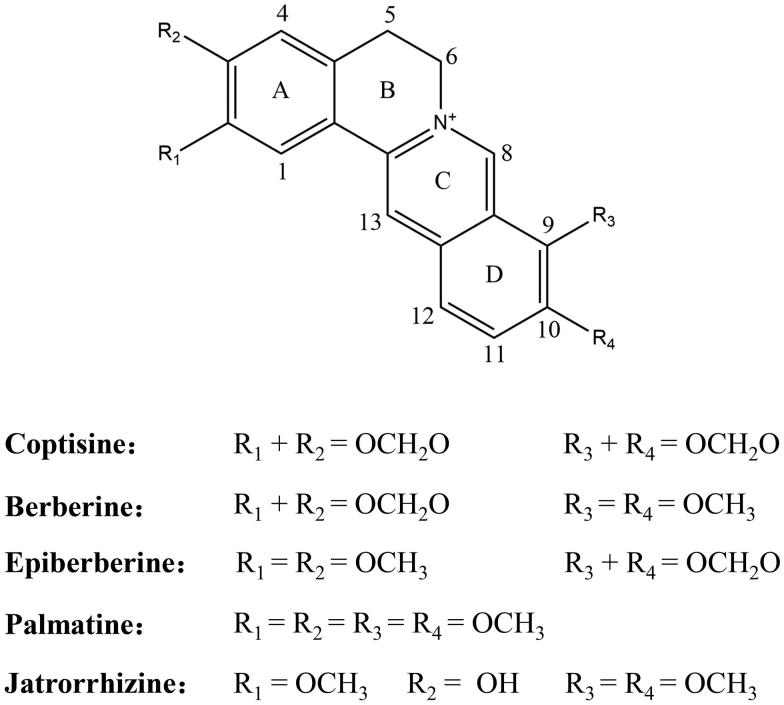
Structures of the five main active alkaloids in Rhizoma Coptidis.

Our previous studies have indeed indicated that Rhizoma Coptidis and its major active alkaloids, palmatine and epiberberine, exhibit excellent anti-*H. pylori* activity and/or inhibit urease, possibly by binding to the urease active site and decreasing the reactivity of active-site thiols in the vicinity of nickel ions^29^^,^^32^^,^^33^. However, berberine fails to show any inhibitory activity against urease^29^. Previous reports indicate that these alkaloids might possess significantly different efficacy in their biological properties, despite their structural similarities. And the characteristic structure of alkaloids seems to be critical to their respective activities^34–37^. Coptisine (structure shown in Figure 1), an active protoberberine alkaloid present in Rhizoma Coptidis, has the same isoquinoline parent structure as alkaloids (berberine, palmatine, epiberberine, and jatrorrhizine) but differs in the appended groups (namely with two methylenedioxy groups). Coptisine exhibits anti-enzyme^38^, antimicrobial^35^, anti-tumour^39^, and gastroprotective effects^40^. However, comparisons of coptisine with other four alkaloids in their biological activities are rarely explored. In this study, we also found that coptisine had the strongest anti-*H. pylori* effects among the five major Rhizoma Coptidis alkaloids. As a follow-up study, we characterised the mechanisms underlying the anti-*H. pylori* activity of coptisine to better understand the anti-*H. pylori* effect of Rhizoma Coptidis alkaloids.

## Materials and methods

### Chemicals and reagents

Berberine, palmatine, coptisine, jatrorrhizine, and epiberberine were obtained from Chengdu Purechem-Standard Co., LTD. (Sichuan, China); all purities were above 98%. Acetohydroxamic acid (AHA; C_2_H_5_NO_2_, purity: 98%), jack bean urease (JBU; type III with activity of 31.66 U/mg solid), urea, dithiothreitol (DTT), L-cysteine (L-cys), glutathione (GSH), sodium fluoride (NaF), and HEPES (Amresco >99%) were purchased from Sigma Aldrich (Steineheim, Germany). Boric acid (BA) was purchased from Aladdin Chemistry Company. Clarithromycin (CLR) and metronidazole (MET) were obtained from Toku-E, Japan. Brain-heart infusion (BHI), Mueller-Hinton agar, and Columbia agar base were obtained from OXOID, USA. Foetal bovine serum (FBS) was purchased from Gibco Rockville, MD, USA. Sheep blood was purchased from Pingrui Biotechnology, China. Other chemicals and analytical reagents were obtained from Guangzhou Chemical Reagent Factory (Guangdong, China). Coptisine stock solution was diluted in 20 mM HEPES (pH 7.5) buffer containing 0.3% dimethyl formamide, which does not affect urease activity at concentrations less than 1%^41^.

### H. pylori strains and preparation of crude H. pylori urease (HPU)

Fourteen *H. pylori* strains were used in this study. NCTC 11637 and NCTC 26695 strains were purchased from American Type Culture Collection. Sydney Strain-1 (SS1) was kindly provided by Prof. Richard Ferrero of Monash University, Australia. Clinical isolate ICDC 111001 was provided by Prof. Zhang Jian-zhong of the Chinese Center for Disease Control and Prevention, China. Ten clinical isolates (Hp1868, Hp1869, Hp1870, Hp1871, Hp1872, Hp1873, Hp1874, Hp1875, Hp1876, and Hp1877) were obtained from Renji Hospital, Shanghai Jiaotong University.

All strains were cultured in Columbia agar base containing 7% sheep blood at 37 °C in a micro-aerophilic environment (5% O_2_, 10% CO_2_, and 85% N_2_). After 3 days, *H. pylori* was scraped and suspended in phosphate buffered saline (PBS). Turbidimetry was employed to adjust the concentrations of different *H. pylori* strains (turbidity equivalent to 1.0 McFarland standard). Crude HPU was obtained according to the method described by Matsubara et al.^42^

### Minimal inhibitory concentration assay

In this study, different concentrations (12.5, 25, 50, and 100 μg/mL) of berberine, palmatine, epiberberine, jatrorrhizine, and coptisine were used to evaluate antimicrobial activity against the standard *H. pylori* strain NCTC 11637. The agar dilution method was then used to analyse the antibacterial effects of coptisine against two *H. pylori* standard strains (SS1 and NCTC 26695) and eleven clinical isolates (ICDC 111001, HP 1868, HP 1869, HP 1870, HP 1871, HP 1872, HP 1873, HP 1874, HP 1875, HP 1876, and HP 1877). In this assay, 100 μL of *H. pylori* suspension (McFarland = 1.0) was added to the test compound-containing or DMSO-containing (control) Columbia blood agar plate. CLR and MET were used as positive controls in this experiment. Minimum inhibitory concentration (MIC) was defined as the lowest concentration at which no strain growth was observed by visual examination after 3 days. This experiment was repeated twice.

### Minimal bactericidal concentration assay

The broth dilution method was used to determine the minimal bactericidal concentrations (MBCs) of coptisine (25, 37.5, 50, 62.5, 75, 100, and 125 μg/mL) against three *H. pylori* standard strains (SS1, NCTC 11637, and NCTC 26695) and eleven clinical isolates (ICDC 111001, HP 1868, HP 1869, HP 1870, HP 1871 and HP 1872, HP 1873, HP 1874, HP 1875, HP 1876, and HP 1877). In brief, *H. pylori* suspension (McFarland = 1.0) was exposed to DMSO (control) and coptisine at 2–4 times MIC in BHI with 10% FBS. After shaking at 120 rpm in the tri-gas incubator for 6 h, 100 μL samples were removed and cultured in Columbia blood agar base^43^. MBC was defined as a 99.9% decrease in viability compared with the control. The experiment was repeated twice.

### pH shock experiment in vivo

*H. pylori* cells (SS1) were harvested, and the concentration was adjusted to Mc = 4 with saline. Then, bacterial samples were mixed with AHA (positive control, 5 mM) or one of the five alkaloids from Rhizoma Coptidis (coptisine, berberine, jatrorrhizine, epiberberine, and palmatine) in 100 mM HCl (pH = 1) or PBS (pH = 7.2) at 37 °C with or without 5 mM urea. After 30 min of incubation, bacteria were collected by centrifugation at 2000*g* for 10 min and resuspended in the same volume of PBS. A dilution series (1:1–1:10000) was made from the samples, and the dilutions were plated on solid agar for 5 days to form single colony. Samples from urea-free wells were used as controls^44^. Results are expressed as CFU/mL; the experiment was repeated in triplicate. Inhibition rate was calculated using the following equation: Inhibition rate = (Urea group bacterial mean − X group bacterial mean)/Urea group bacterial mean ×100%, where X represents each experimental group.

### Measurement of standard urease activity

This assay was performed using the modified Berthelot method (phenol-hypochlorite) in 96 well plates^45^. At the beginning of each reaction, different concentrations of urease solution and 150 mM urea as a substrate were mixed in 20 mM HEPES buffer (pH 7.5) at 37 °C. After a 20-min reaction, urease activity was tested by measuring the concentration of ammonia produced by the reaction at 595 nm using the microplate reader (Thermo Fisher Multiskan FC, Instruments, Inc., USA) at ambient temperature. The activity of HPU was determined to be 17.0 U/mg by comparing it to JBU, which had a known activity of 31.66 U/mg solid. One unit (U) of urease activity was defined as the amount of urease required to catalyse 1 μmol urea per min. These experiments were repeated in triplicate.

### Urease inhibition assay *in vitro*

Coptisine’s ability to inhibit urease activity was determined by measuring amounts of ammonia produced using the modified Berthelot method described above. The assay mixture, which contained 200 μL of enzyme and 200 μL of coptisine solution (0.16–2.51 mM) in 200 μL of 20 mM HEPES buffer (pH 7.5), was incubated for 20 min at 37 °C in 96-well plates. The reaction was then initiated by adding urea solution (150 mM) to determine residual urease activity. AHA was used as a standard urease inhibitor. Urease activity in the absence of inhibitors was used as a positive control to define 100% activity. Residual activity (RA %) for each assay was calculated using the following equation: RA % = (activity with inhibitors/activity without inhibitors) × 100%. The assay was repeated in triplicate.

### Determination of urease inhibition type

Using the methods described above, the type of enzyme inhibition induced by coptisine was determined by examining changes in the Michaelis constant (*K*_M_) and maximum velocity (*v*_max_) values from Lineweaver–Burk plots of 1/*v* versus 1/urea. The inhibition constant was determined using secondary plots of the apparent *K*_M_/*v*_max_ or 1/*v*_max_ versus the concentration of coptisine. The assays were performed in triplicate.

### Progress curve analysis

Reaction progress curves for urea hydrolysis in the non-pre-incubated and pre-incubated systems were generated by plotting the concentration of ammonia generated as a function of the incubation time *P*(*t*) in the absence or presence of coptisine. Coptisine and urease were mixed immediately before the reaction in the non-pre-incubated system and were mixed and incubated for 20 min before urea was added to initiate the reaction in the pre-incubated system. Urease activity relative to the standard assay was determined at different time intervals.

### Effects of thiol compounds on coptisine-modified urease

Different incubation times and orders of component addition were used to examine the effects of thiol-containing compounds (1.25 mM DTT, GSH, or L-cys, freshly prepared) on urease inhibited by coptisine. Briefly, mixtures of urease, thiol compounds, and coptisine (2.5 mM) were incubated at 37 °C for different amounts of time (5–30 min). Additionally, two of the reactants (urease, thiol compounds, and coptisine) were pre-incubated at 37 °C for 20 min followed by addition of the third compound and 20 additional min of incubation in separate assays. Urease activity was measured relative to the standard assay.

### Effects of inorganic reagents on coptisine-modified urease

BA and NaF were used to analyse the influence of inorganic reagents on urease in the presence and absence of coptisine. Briefly, coptisine was added to a mixture containing urease and the inorganic reagent (5 mM BA or NaF) after a 20-min co-incubation at 37 °C. After another 20-min co-incubation, the mixture was added to the standard assay system and absorbance was measured at 595 nm. The assay was repeated three times.

### Remobilisation of coptisine-inhibited urease

The ability of DTT (freshly prepared) to reactivate coptisine-inhibited urease was investigated in this assay. Urease was pre-incubated with coptisine until enzyme activity decreased to approximately 20% of baseline. DTT (1.25 mM) was then added and incubated with the coptisine-deactivated urease for different amounts of time. Urease activity before and after the addition of DTT was measured using the standard assay.

### UV-visible spectroscopy

Binding of Ni^2+^ and thiols to coptisine was examined using UV-visible spectroscopy on a TU-1810PC UV-visible spectrophotometer (Purkinje, Beijing, China) at ambient temperature (∼25 °C). UV-visible spectra were scanned from 200 to 600 nm. For Ni^2+^ and L-cys titration experiments, coptisine solutions were prepared in a titration buffer (20 mM HEPES, pH 7.5) with or without different concentrations of nickel chloride and L-cys. The spectrum obtained from buffer without nickel chloride or L-cys was used for baseline correction.

### Molecular docking study

The molecular docking study was performed as previously described^46^. AutoDock version 4.2 and the AutoDock Tools (ADT 1.5.6) graphic workstation were used to analyse the mode and free energy values of binding between urease and coptisine. The three-dimensional structures of JBU (PDB ID: 3LA4, resolution: 2.05 Å) and HPU (PDB ID: 1E9Y, resolution: 3.00 Å) were downloaded from the RCSB Protein Data Bank, and the energy-efficient three-dimensional shape of coptisine was examined using chem3D Ultra 8.0 software. A grid map was generated to evaluate the possible binding sites of coptisine on urease. The size of the cubic grid box was set to 60 Å (*x*, *y*, and *z*) with a spacing of 0.375 Å, and the midpoint of the two nickel ions was defined as the centre of the grid box. Before the initiation of the experiment, water molecules were removed from and polar hydrogen atoms were added to urease using AutoDock tools. Binding free energy was computed using the Lamarckian genetic algorithm (LGA)^47^; coptisine–urease complexes with the lowest free energy of combination were considered the most favourable structures. Distances (Å) between hydrogen bond-forming residues were also measured.

### UreG activity inhibition assay

UreG was expressed and purified as described previously^26^. Briefly, the UreG expression vector was constructed by sub-cloning the *ureG* gene into an in-house pHisSUMO vector, which contains the coding sequence of a HisSUMO tag on a pRSETA vector (Invitrogen). UreG was expressed as a HisSUMO-tagged fusion protein using transformed *Escherichia coli*. HisSUMO-UreG-expressing bacterial cells were lysed via sonication in buffer B (20 mM Tris/HCl, pH 7.5, 200 mM NaCl, 1 mM TCEP, and 40 mM imidazole) and loaded onto a 5 mL HisTrap column (GE Healthcare) equilibrated with buffer B. After washing with 10 column volumes of buffer B, HisSUMO-UreG was eluted with 300 mM imidazole in buffer B. The HisSUMO tag was cleaved using small ubiquitin-like modifier protease SENP1C and separated from UreG by a second pass through the HisTrap column. UreG was further purified by size exclusion chromatography using HiLoad 26/60 Superdex 75 columns (GE Healthcare) in buffer B without imidazole. Purified UreG was dialysed again with 5 mM EDTA to remove any bound metal before further experimentation.

The assay was performed in 96-well plate. Briefly, 100 μM GTPγS (positive control) or coptisine was added to the reaction buffer containing 5 μM UreG monomer, 2.5 μM NiSO_4_, and 5 mM NaHCO_3_. 300 μM GTP was then added after 60, 48, 36, 24, or 12 min to start the reaction at 37 °C. Buffer A (150 mM KCl, 2 mM MgSO_4_, 100 mM Tris, pH adjusted to 7.5 with HCl) was added to bring the final reaction volume to 200 µL. At the end of the reaction, 50 μL malachite green solution was added to measure the amount of phosphate produced in the reaction^48^, and the plate was read at 630 nm after 5 min of incubation at room temperature. Relative activity values were calculated compared to UreG group (drug-free) reactions using 100 µM coptisine or GTPγS. The IC_50_ value was then calculated based on GTP hydrolysis rates in the presence of coptisine at concentrations of 1.25–500 μM using KaleidaGraph 4.0.

### Fluorescence emission spectra of coptisine titration with GTP

To determine whether coptisine affected GTP during coptisine-induced UreG inhibition, the interaction between GTP and coptisine was monitored using fluorescence emission spectra. Fluorescence data were collected on a SpectraMax i3x multimode plate reader (Molecular Devices, Sunnyvale, CA, USA). Coptisine (500 μM) was dissolved in buffer A (150 mM KCl, 2 mM MgSO_4_, and 100 mM Tris, pH 7.5) in the absence or presence of GTP at the desired concentrations (100, 200, 300, 400, or 500 μM). The solutions were incubated at 37 °C in the dark for 60 min before fluorescence measurement. The excitation wavelength was 380 nm.

### UreG dimer formation inhibition assay

Size exclusion chromatography technology was used to analyse the effects of coptisine on UreG dimer formation. Experiment was carried out in the buffer containing 5 μM UreG monomer and 2.5 μM NiSO_4_, and divided into the following three groups: 300 μM GTP (positive control), buffer A (negative control), and 300 μM GTP +500 μM coptisine. After reaction for 60 min, samples were then injected into a Superdex 75 analytical gel filtration column pre-equilibrated with buffer A.

### Nickel uptake assay

UreG dimers were obtained using GTPγS as described previously^26^. Briefly, the UreG dimer was purified by incubating apo-UreG at 3 mg/mL on ice with 1 mM GTP, 2 mM MgSO_4_, and 0.5 mM NiSO_4_ for 15 min. Nickel-charged UreG dimers were then separated from excess GTP and nickel using a Sephadex G25 desalting column. UreG dimers (2.5 μM) were injected into dialysis bag (6–8 kDa) and mixed with different concentrations of coptisine (25, 50, or 100 μM) or DMSO (control). The dialysis bag was sealed and placed in beakers containing UreG dimer buffer at 37 °C in a water bath. After 30 min, dialysis bags were placed in fresh buffer and the sample was incubated for 2 h at 4 °C with continuous shaking to initiate dialysis. The dialysis process was repeated three times. Samples were then removed and nickel concentrations were measured using atomic absorption spectroscopy (AA-7000, SHIMADZU). Nickel concentration was determined by comparing measurements against a standard curve of known nickel concentrations.

### Statistical analysis

Statistical analysis was performed with GraphPad Prism 5 (GraphPad Software, Inc.) and SPSS 13.0 (SPSS, Inc.) softwares. Data were shown as means ± standard deviation (SD). Statistical differences between groups were calculated by one-way analysis of variance (ANOVA) followed by Dunnett's test. Statistical probability of 0.05 or *p* < 0.01 was considered significant.

## Results

### MIC of Rhizoma Coptidis alkaloids against *H. pylori*

An antibacterial study revealed that the main alkaloids in Rhizoma Coptidis inhibited growth of the standard *H. pylori* strain NCTC 11637 at pH 7.2 to varying degrees (Table 1). Coptisine, with an MIC of 25 μg/mL, had a two to fourfold more potent anti-*H. pylori* effect than the other four alkaloids tested (berberine, palmatine, epiberberine, and jatrorrhizine). In comparison, the standard antibacterial agents, CLR and MET exhibited appreciable anti-*H. pylori* activity, with MICs of 2 × 1.0^−2^ and 50 × 1.0^−2 ^μg/mL, respectively.

**Table 1.** MICs of the main alkaloids in Rhizoma Coptidis and the reference drugs against *H. pylori* standard strain (NCTC 11637).

Compounds	MIC (µg/mL)
Coptisine	25
Berberine	50
Palmatine	100
Epiberberine	100
Jatrorrhizine	100
CLR	2 × 10^−2^
MET	50 × 10^−2^

*Note*. CLR, clarithromycin; MET, metronidazole; MIC, minimum inhibitory concentrations.

### MICs and MBCs of coptisine against *H. pylori*

As shown in Table 2, MICs of coptisine against the three standard *H. pylori* strains SS1, NCTC 26695, and NCTC 11637 were all 25 μg/mL, while the MICs of coptisine against eleven clinical isolates obtained from three sites in China were either 25 or 50 μg/mL. Moreover, the MBCs of coptisine against the three *H. pylori* standard strains and eleven clinical isolates ranged from 37.5 to 125 μg/mL. An agent is considered bactericidal if the MBC is not more than four times the MIC^49^. The MBC/MIC values for coptisine ranged from 1.25 to 2.5, indicating that coptisine exhibited bactericidal effects against *H. pylori*. Importantly, coptisine showed potent antibacterial and bactericidal effects against isolates with CLR resistance (HP 1870 and HP 1876) and MET resistance (HP 1870, HP 1876, HP 1869, and HP 1871)^43^, with MICs of 25, 50, 50, 25 μg/mL, and MBCs of 37.5, 75, 62.5, 37.5 μg/mL, respectively.

**Table 2.** MICs and MBCs of coptisine against different *H. pylori* strains under pH = 7.2.

Strains	MIC (µg/mL)	MBC (µg/mL)	MBC/MIC
SS1	25	37.5	1.5
NCTC 26695	25	37.5	1.5
NCTC 11637	25	62.5	2.5
ICDC 111001	25	50	2
HP 1868	50	62.5	1.25
HP 1869	50	62.5	1.25
HP 1870	25	37.5	1.5
HP 1871	25	37.5	1.5
HP 1872	25	37.5	1.5
HP 1873	50	75	1.5
HP 1874	50	100	2
HP 1875	50	75	1.5
HP 1876	50	75	1.5
HP 1877	50	125	2.5

### Coptisine inhibits urea-dependent survival of *H. pylori* under acidic conditions

After the pH shock (incubation at pH 1 for 30 min), 472 ± 57 CFU/mL of *H. pylori* survived in the medium with 5 mM urea (Table 3), while the bacteria did not survive without the addition of urea (control). On the other hand, coptisine (50, 100, and 200 μM) potently inhibited the urea-dependent survival of *H. pylori* under acidic (pH = 1), but not neutral (pH = 7.2), conditions in a dose-dependent manner, with inhibition rates of 72.7%, 94.7%, and 98.5%, respectively. In comparison, AHA, a well-known urease inhibitor^50^, resulted in 100% inhibition under acidic, but not neutral, conditions. However, the other Rhizoma Coptidis alkaloids (berberine, palmatine, epiberberine, and jatrorrhizine) failed to inhibit *H. pylori* after 30 min under either acidic or neutral conditions, even at concentrations of 100 μM. These results suggest that the anti-*H. pylori* effect of coptisine *in vivo* is a result of urease inhibition.

**Table 3.** Effect of the main alkaloids from Rhizoma Coptidis on urease activity *in vivo* (*n* = 3).

Groups (μM)	pH =1		pH =7.2	
	CFU/mL	Inhibition rate (%)	×10^4^ CFU/mL	Inhibition rate (%)
Control	0 ± 0	–	1.1 ± 0.3	–
Urea	472 ± 57	–	1.3 ± 0.2	–
Acetohydroxamic acid	0 ± 0	100	1.7 ± 0.4	0
Coptisine (50)	129 ± 31	72.7	1.0 ± 0.2	0
Coptisine (100)	25 ± 6	94.7	1.3 ± 0.3	0
Coptisine (200)	7 ± 3	98.5	1.2 ± 0.3	0
Berberine (100)	587 ± 103	0	1.2 ± 0.4	0
Jatrorrhizine (100)	552 ± 98	0	1.2 ± 0.3	0
Epiberberine (100)	481 ± 92	0	1.3 ± 0.3	0
Palmatine (100)	628 ± 110	0	1.2 ± 0.4	0

*Note*. “0” indicates absence of urease inhibition effect. And “–” means no calculation.

### Coptisine inhibits urease activity *in vitro*

**Figure 2.**
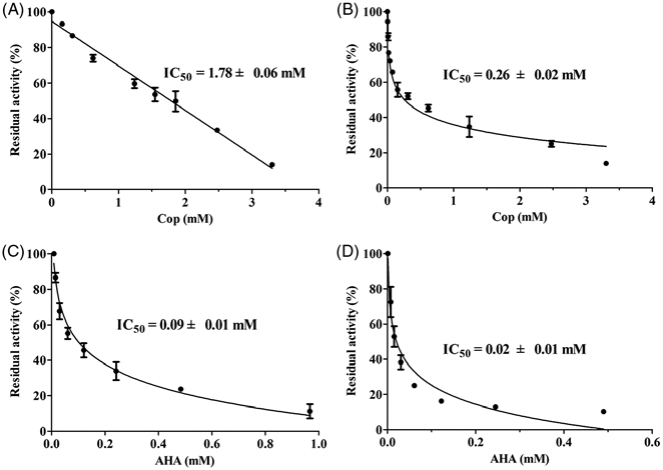
Coptisine (Cop)-induced inhibition of HPU (A) and JBU (B). Effects of AHA on HPU (C) and JBU (D). Results are expressed as means ± SD of three independent experiments.

As shown in Figure 2, coptisine-induced inhibition of urease was dose-dependent, with IC_50_ values of 1.78 ± 0.06 and 0.26 ± 0.02 mM for HPU (Figure 2(A)) and JBU (Figure 2(B)), respectively. The IC_50_ values for AHA, the positive control, were 0.09 ± 0.01 and 0.02 ± 0.01 mM for HPU (Figure 2(C)) and JBU (Figure 2(D)), respectively.

### Inhibition type analysis

**Figure 3.**
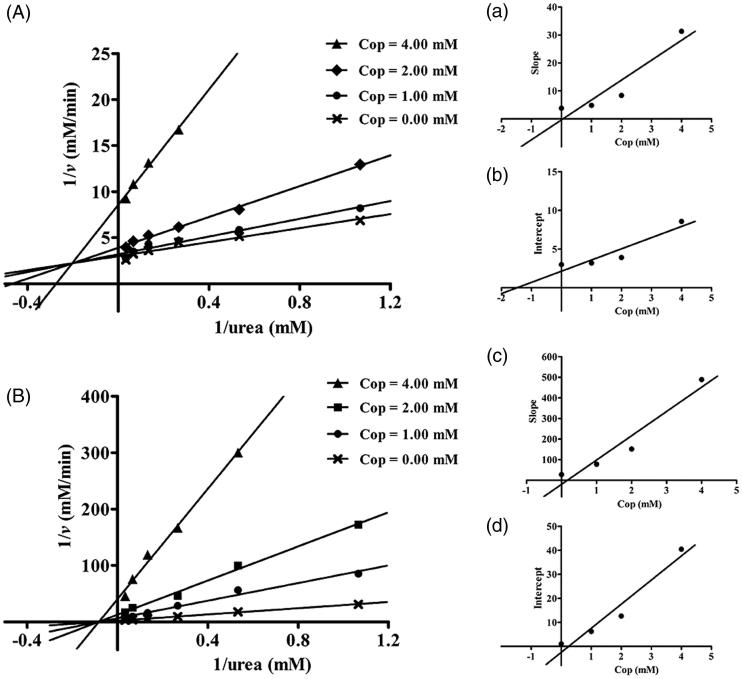
Determination of inhibition type and inhibition constant of coptisine (Cop) on urease. Lineweaver–Burk plots of 1/v versus 1/urea in HPU (A) and JBU (B) in the absence or presence of 1.00, 2.00, or 4.00 mM Cop. (a, c) The inhibition constant Ki was determined from the plot of slope versus Cop concentration. (b, d) The inhibition constant Kis was determined from the plot of intercept versus Cop concentration.

The mechanism by which coptisine inhibits urease was investigated by measuring variations in the Michaelis constant (*K*_M_) and maximum velocity (*v*_max_) values using Lineweaver–Burk plots. Several straight lines intersected at the same point in the second quadrant, while *K*_M_ increased and *v*_max_ decreased gradually after the addition of various concentrations of coptisine, suggesting that coptisine was a mixed-type inhibitor for HPU (Figure 3(A)). Furthermore, the equilibrium constants for binding of coptisine to the free enzyme (*K_i_*) and the enzyme substrate complex (*K_is_*) were 0.05 and 1.48 mM, respectively, and were calculated based on the slope or the vertical intercept versus the inhibitor concentration, respectively (Figure 3(A) and (B)).

The plot of 1/*v* versus 1/urea consisted of several straight lines that intersected each other at the same point on the X-axis, suggesting that the value of *K*_M_ did not change much, while the *v*_max_ value gradually decreased, as the coptisine concentration increased for JBU (Figure 3(B)). This might indicate that coptisine non-competitively inhibits JBU. The inhibition constant *K_i_* and *K_is_* values were 0.17 and 0.25 mM, respectively (Figure 3(C) and (D)).

### Reaction progress curves

**Figure 4.**
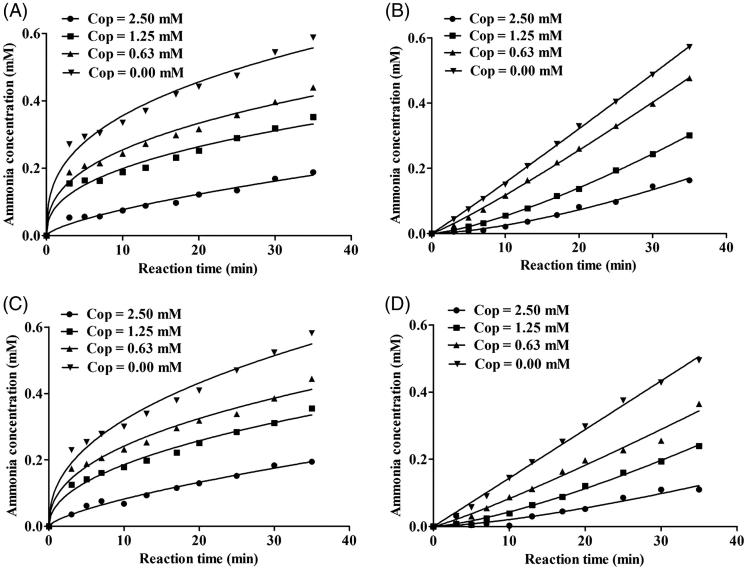
Reaction progress curves of urease-catalysed hydrolysis of urea in the presence or absence of coptisine (Cop). Reaction progress curves in the non-pre-incubated (A: HPU; B: JBU) and pre-incubated systems (C: HPU; D: JBU). Curves were obtained by measuring concentration of ammonia versus incubation time. The concentrations of Cop were 0, 0.63, 1.25, and 2.50 mM.

As shown in Figure 4, inhibitor concentration and incubation time had obvious effects on binding rate between coptisine and HPU and JBU. Furthermore, there were no significant differences between the non-pre-incubated system and the pre-incubated system. The reaction progress curves for HPU and JBU were consistent with the slow-binding inhibition characterised in a previous report^51^. A curve-fitting programme was used for these data points to measure progress of the slow-binding reaction according to the equation: *P*(*t*) = *v_s_* × *t* + (*v*_0_ − *v_s_*) (l − *e*^−^*^k^*^app ×^*^t^*) *k*_app_^−1^, where *P*(*t*) is the amount of accumulated product at time *t*, *v*_0_ and *v_s_* are the initial and steady-state reaction velocities, respectively, and *k*_app_ denotes the apparent velocity constant.

As shown in Figure 4(A), reaction progress for coptisine-HPU binding exhibited a characteristic concave downward shape, indicating that binding involved the rapid formation of a collision complex (EI) then which underwent a slow conversion into a more stable final complex EI*. The initial velocity (*v*_0_) of urea hydrolysis decreased to a much slower steady-state velocity (*v_s_*) according to the apparent first-order velocity constant *k*_app_. However, in the reaction system with coptisine and JBU, the linear curves demonstrated that the equilibrium among the enzyme, inhibitor and EI* complex was characterised by the steady-state velocity (*v_s_*) (Figure 4(B)).

### Thiol-containing compounds protect urease against coptisine-induced inhibition

**Figure 5.**
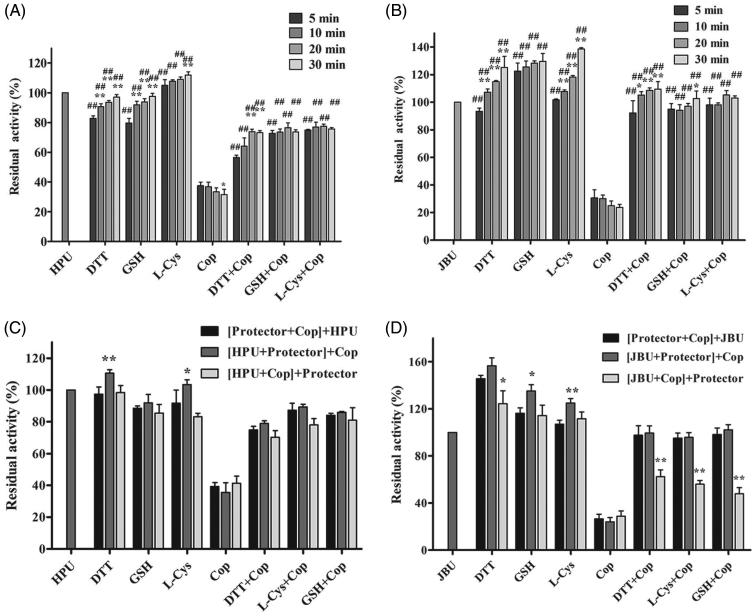
Effects of incubation time and order of component addition on coptisine (Cop)-modified HPU (A and C) and JBU (B and D). Urease activity was measured after incubation for 5, 10, 20, or 30 min. The compounds in brackets were pre-incubated for 20 min, and an additional 20-min incubation was performed after the last compound (outside of the brackets) was added. 1.25 mM thiol-containing compound and 2.5 mM Cop were used. 1.25 mM thiol compound without coptisine served as controls. Results are shown as means ± SD of three independent tests. One-way ANOVA revealed significant differences compared to the first column for each group and the Cop alone treatment group; *p < 0.05, **p < 0.01, and ##*p* < 0.01.

Thiol-containing compounds exert protective effects by interacting with the sulfhydryl group (–SH) of urease to restrict the ability of inhibitors to access the active site^52,53^. We therefore performed an assay to investigate the potential effects of thiol-containing compounds (DTT, L-cys, and GSH) on coptisine-treated urease. As shown in Figure 5, residual enzyme activity of coptisine-treated urease was higher in the presence of thiol-containing compounds than in the thiol-free system, suggesting that the thiol compounds had protected urease against coptisine-induced inhibition. Furthermore, this protective effect was stronger for JBU than for HPU. However, the incubation time had limited effects in this urease activity assay (Figure 5(A) (HPU) and 5(B) (JBU)). Furthermore, while the order in which urease, inhibitor, and thiols were added did not significantly affect HPU (Figure 5(C)), residual enzyme activity was the lowest for JBU when thiols were added after coptisine (Figure 5(D)). Thus, inhibition of urease by coptisine might involve binding with the active site sulfhydryl group.

### Effects of inorganic reagents on coptisine-modified urease

**Figure 6.**
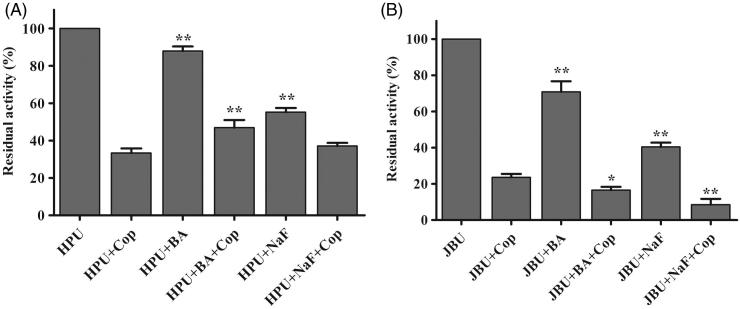
Effects of inorganic compounds on coptisine (Cop)-treated urease. 2.5 mM Cop and 5 mM inorganic compound (BA or NaF) were used. Results are expressed as means ± SD of three replicates. One-way ANOVA revealed significant differences compared with the Cop alone treatment group; *p < 0.05, **p < 0.01.

Studies have shown that the inorganic compounds BA and NaF, two well-known competitive urease inhibitors, inhibit urease activity by binding to the nickel ions (Ni^2+^) in urease^19, 54^. Here, coptisine, NaF, and BA exhibited varying suppressive effects on HPU and JBU, and coptisine was the strongest urease inhibitor. Moreover, residual HPU activity was higher, whereas residual JBU activity was lower, in the mixed system containing urease, inorganic reagents, and coptisine compared to coptisine without inorganic reagents (Figure 6(A) and (B)). This result suggested that the inorganic compounds (BA and NaF) had a protective effect on HPU against coptisine inhibition, which indicated that the binding of coptisine to the active site might involve interactions with the Ni^2+^ ion. In contrast, a synergic effect was observed for JBU, suggesting that the inhibitory mechanism might involve blockage of its sulfhydryl group.

### UV-visible spectroscopy

**Figure 7.**
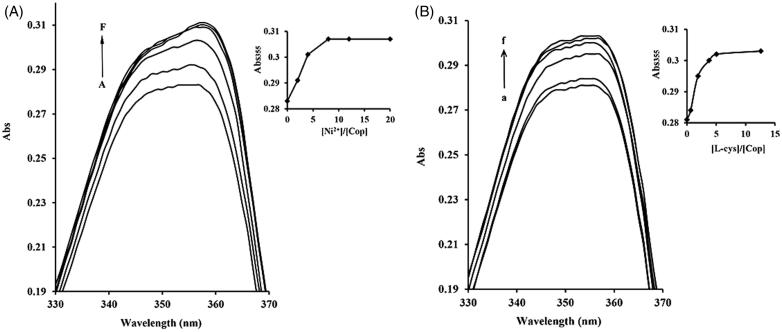
Ni^2+^ and L-cys binding to coptisine (Cop) measured using UV-visible spectroscopy. 16 μM Cop in the absence or presence of different molar equivalents of Ni^2+^ (1 mM NiCl_2_) (A) or L-cys (1 mM) (B) in 20 mM HEPES buffer, pH 7.5. A-F, 0.0 to 320 μM of Ni^2+^; A-F, 0.0 to 200 μM of L-cys. The inset shows the titration curves for binding of Ni^2+^ or L-cys to Cop at 355 nm. Wavelength (nm) is represented on the *x*-axis and absorption (Abs) is represented on the *y*-axis.

The most pivotal catalytic components of ureases are the cysteinyl residues and Ni^2+^ ions located in the active site. We therefore examined whether coptisine affected Ni^2+^ and L-cys using UV-visible spectroscopy. The maximum optical properties of bands at ∼355 nm for coptisine increased with the further addition of Ni^2+^ and L-cys, and levelled off at a Ni^2+^: coptisine molar ratio of 8.0 and a L-cys: coptisine ratio of almost 5.0, indicating that Ni^2+^ and L-cys differentially bind to coptisine (Figure 7(A) and (B)).

### Reactivation of coptisine-inhibited urease

**Figure 8.**
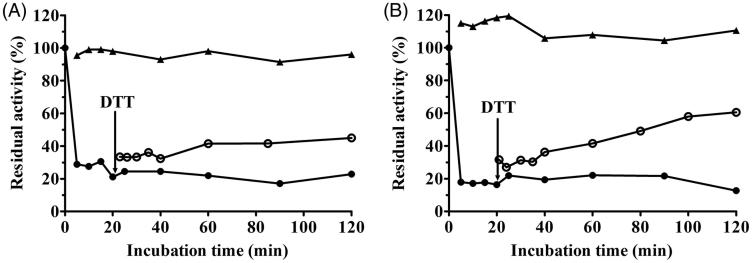
Remobilisation of coptisine (Cop)-deactivated HPU (A) and JBU (B) by 1.25 mM DTT. Urease activity was inhibited by 3.0 mM Cop (•) and was restored by the addition of DTT (^) after 20 min. DTT (▲) without Cop served as the control.

A DTT assay was performed to further explore the effect of sulfhydryl compounds on urease and the stability of the urease–coptisine complex. Administration of 1.25 mM DTT without coptisine served as a control. As shown in Figure 8, activity of coptisine-inhibited urease was at least partially restored by 1.25 mM DTT, increasing from 20% of its initial activity after a 20-min incubation to nearly 45% and 60% after 120 min for HPU and JBU, respectively. This result revealed that coptisine-induced inhibition of urease was reversible. This restoration of activity confirmed the results of the urease protection experiment with thiol-containing reagents above, further indicating that the sulfhydryl groups in the urease active site are crucial to the inactivation of urease by coptisine.

### Molecular docking study

**Figure 9.**
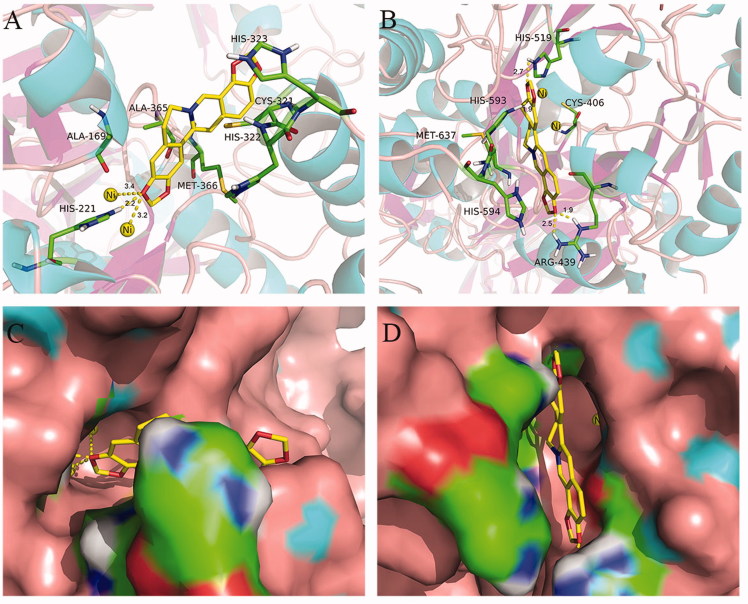
Interactions between coptisine (Cop) and HPU (A) and JBU (B) in molecular docking simulations. Ni2+ ions are represented by small yellow spheres. Surface representation of the active site flap of HPU (C) and JBU (D) in the presence of coptisine (colours represent atoms; carbon is yellow, nitrogen is blue, oxygen is red, hydrogen is grey, sulphur is orange. Yellow dashes represent hydrogen bonds between the receptor and ligand).

We used an automated molecular docking simulation to investigate potential binding modes between coptisine and HPU and JBU. Mean free energy of binding values, which were calculated using the LGA, were −6.60 kcal/mol for HPU and −6.86 kcal/mol for JBU. Optimal binding modes for coptisine are shown in both cartoon form (Figure 9(A) and (B)) and in enzyme surface depictions (Figure 9(C) and (D)). For HPU (Figure 9(A) and (C)), the coptisine 3-O-CH_2_- group formed hydrogen bond interactions with the NH group of His-221 at a distance of 2.2 Å and metal-ligand with the two nickel ions at distances of 3.2 and 3.4 Å, respectively. The benzene rings of coptisine might form hydrophobic interactions with Ala-169, Ala-365, Met-366, His-322, Cys-321, and His-323.

For JBU, autodocking results suggested that coptisine tightly anchored the helix-turn-helix motif over the active site cavity through N-H⋯O hydrogen bonding interactions, which prevented the mobile flap from returning to the closed position. Briefly, the coptisine 10-O-CH_2_- group interacted with both NH groups of Arg-439 at distances of 1.9 and 2.5 Å, respectively. The coptisine 3-O-CH_2_- and 2-O-CH_2_- groups also formed hydrogen bonding interactions with the NH groups of His-519 and His-593 at distances of 2.7 and 1.9 Å, respectively. Additionally, the isoquinoline ring of coptisine might form hydrophobic interactions with Met-637, His-594, and Cys-406 (Figure 9(B) and (D)).

### Coptisine inhibits UreG activity

**Figure 10.**
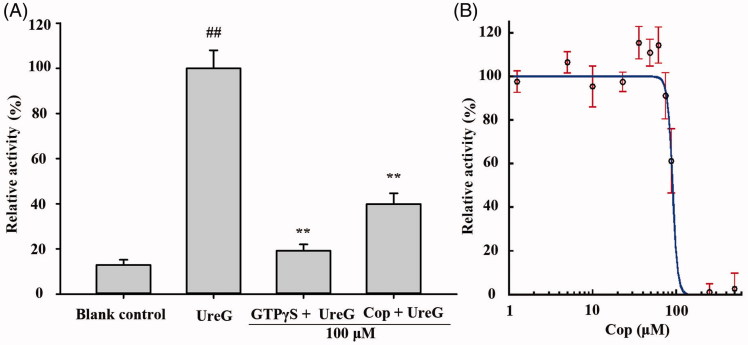
Coptisine (Cop) inhibits UreG activity. (A) 100 μM GTPγS (positive control) or Cop (100 μM) was added to reaction buffer containing 5 μM UreG monomer, 2.5 μM NiSO4, and 5 mM NaHCO3. 300 μM GTP was then added after 60, 48, 36, 24, or 12 min to start the reaction at 37 °C. The group without drug (GTPγS or Cop) was labelled UreG, while the group without UreG monomer and drug was designated the blank control. UreG activity in the absence of inhibitors was defined as 100% activity for comparison. (B) The IC50 value of coptisine was calculated using the initial GTP hydrolysis rate of Cop at concentrations ranging from 1.25 to 500 μM. Results are shown as means ± SD of triplicate tests. UreG activity in the absence of inhibitors was used to define 100% activity for comparison. ##*p* < 0.01 and **p < 0.01 suggested significant differences compared with blank control and UreG, respectively.

Urease maturation in *H. pylori* involves nickel delivery to the metallo-active centre and is highly dependent on the cooperation of at least four urease accessory proteins (UreE, UreG, UreF, and UreH). Among these, UreG plays a vital role as a nickel chaperone and GTPase enzyme during urease maturation. A previous experiment has demonstrated that nickel induces GTP-dependent dimerisation of UreG, and the UreG dimer can be hydrolysed in the presence of bicarbonate, resulting in phosphate release^26^. As shown in Figure 10(A), the UreG dimerisation reaction was inhibited by 100 μM coptisine, with an IC_50_ value of 89.86 ± 3.26 μM (Figure 10(B)), indicating that coptisine could inhibit the function of prototypical accessory protein UreG during urease maturation.

### Fluorescence spectroscopy

**Figure 11.**
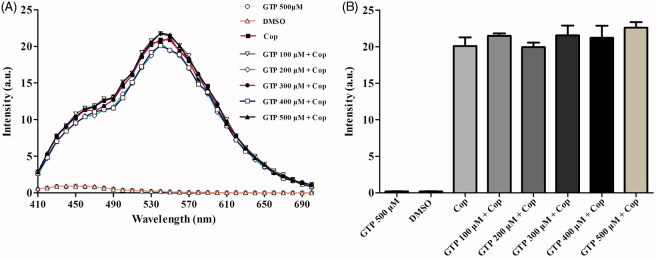
Fluorescence emission spectra of coptisine (Cop) in different concentrations of GTP solution. (A) Cop (500 μM) was dissolved in buffer A (150 mM KCl, 2 mM MgSO4, and 100 mM Tris, pH 7.5) in the absence or presence of GTP at the desired concentration (100, 200, 300, 400, or 500 μM). The solutions were incubated at 37 °C for 60 min before fluorescence measurement. The excitation wavelength was 380 nm. (B) Maximum fluorescence intensity in the absence or presence of various concentrations of GTP was measured at 540 nm.

Fluorescence spectroscopy was used to investigate potential interactions between coptisine and GTP. Briefly, coptisine was incubated with different concentrations of GTP, and resulting fluorescence was measured using a fluorescence microplate. As shown in Figure 11, the intensities of the coptisine fluorescence emission spectra were independent of GTP concentration, and the maximum emission peak was observed at ∼540 nm. The results suggest that coptisine has no influence on GTP.

### Coptisine inhibits UreG dimer formation

**Figure 12.**
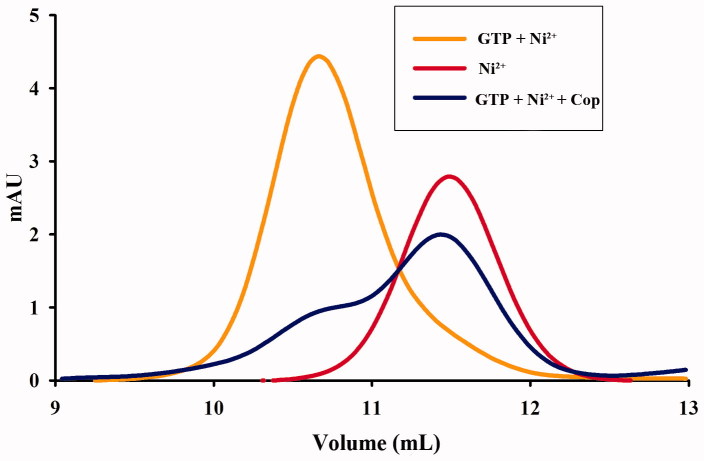
Coptisine (Cop) inhibits formation of UreG dimers. UreG monomers were treated with GTP + Ni^2+^ (orange), Ni^2+^ (GTP-free, red), or GTP + Ni^2+^ + Cop (blue).

Nickel and GTP induce the formation of nickel-charged UreG dimers, which are composed of two UreG monomers and one nickel ion. We therefore examined the effect of coptisine on UreG dimer formation. As shown in Figure 12, UreG dimers (GTP + Ni^2+^) and UreG monomers (Ni^2+^) were observed in two distinct peaks that differed approximately twofold in molecular weight. UreG dimerisation was obviously inhibited by treatment with 500 μM coptisine, suggesting that coptisine interferes with urease maturation by blocking UreG dimer formation.

### Coptisine promotes dissociation of nickel from UreG dimers

**Figure 13.**
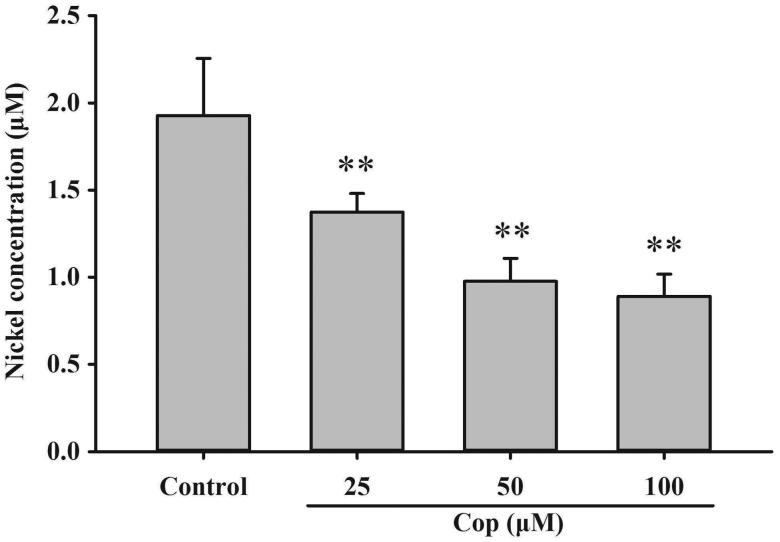
Coptisine (Cop) inhibits nickel ion transfer from UreG dimers. UreG dimer (2.5 μM) was mixed with different concentrations of coptisine (25, 50, and 100 μM) and DMSO (control). Results are shown as means ± SD of triplicate tests. **p < 0.01 compared to the control group.

The urease active site contains two nickel ions that are necessary for catalysis. To become enzymatically active, apo-urease undergoes post-translational carboxylation of an active site lysine residue followed by the insertion of nickel ions. The UreG dimer plays a key role in the delivery of nickel ions during urease maturation. An assay was performed to examine the effect of coptisine on nickel delivery and UreG dimer function. Coptisine (25, 50, and 100 μM) markedly decreased bound nickel concentrations of UreG dimers in a dose-dependent manner during the dialysis process compared to the control (all *p* < 0.01). This observation indicates that coptisine promotes the dissociation of nickel from UreG dimers (Figure 13).

## Discussion

Coptisine, an alkaloid found in Rhizoma Coptidis, possesses the same tetracyclic structure as alkaloids (berberine, epiberberine, palmatine, and jatrorrhizine) but contains different substituents on ring A or ring D, namely two methylenedioxy groups. However, comparisons of the biological activities of coptisine and other four alkaloids are lacking. In the present study, the inhibitory effects of coptisine on *H. pylori* and the potential underlying mechanisms were investigated.

MIC and MBC assays showed that coptisine exhibited varying antibacterial and bactericidal effects towards all tested *H. pylori* strains, including three reference strains (NCTC 26695, NCTC 11637, and SS1) and eleven clinical isolates (including four antibiotic-resistant strains). Among the five alkaloids tested, MIC values indicated that coptisine inhibited *H. pylori* to the greatest degree, followed by berberine, epiberberine, palmatine, and jatrorrhizine. However, another study found that berberine was the most effective at inhibiting *Bacillus shigae* and *E. coli*, followed by coptisine and palmatine^55^^,^^56^.

All five of the tested alkaloids share the same isoquinoline parent structure characterised by quaternary nitrogen and aromaticity, which might contribute to their anti-*H. pylori* activities. However, the specific groups located in the A (C2 and C3) and D (C9 and C10) rings, which differ among the alkaloids, seemed to play a more crucial role in their anti-*H. pylori* activities. Indeed, each alkaloid was crucial to their respective activities based on its characteristic structure^36^. The biological activities of structurally related isoquinoline alkaloids of protoberberine class exhibited significant differences, such as vascular smooth muscle cell inhibition^36^, hydroxyl radical scavenging activities^34^, antimicrobial activity^35^, anti-tumour activity^37^ and so on. Our finding that coptisine has the strongest anti-*H. pylori* effect indicates that its methylenedioxy functional groups in the A and D rings, which are more lipophilic than methoxy groups, may be crucial for enhanced *H. pylori* inhibition. However, further investigation of larger numbers of samples should be performed to confirm this structure-activity relationship.

Urease, which neutralises the acidic environment of the human stomach, is essential for the survival and pathogenesis of *H. pylori*. Previous studies have shown that coptisine inhibits the activity of various enzymes, such as elastase^57^, acetylcholinesterase^38^, CYP2D6^58^, indoleamine 2,3-dioxygenase^59^, aldose reductase^60^, monoamine oxidase^61^, and bacterial collagenase^62^. Here, the pH shock experiment revealed that coptisine potently inhibited *H. pylori* under acidic, but not neutral, conditions within 30 min, suggesting that coptisine likely inhibited urease activity. In contrast, berberine, palmatine, epiberberine, and jatrorrhizine did not effectively inhibit *H. pylori* under either acidic or neutral conditions (Table 3).

The *in vitro* assay indicated that coptisine inhibited both HPU and JBU in a dose-dependent manner. We previously found that epiberberine and palmatine inhibited urease activity *in vitro* more effectively than coptisine^32^^,^^33^. However, *H. pylori* both contains internal (intracellular) and secretes external (extracellular) ureases, and internal urease in particular is vital for acid neutralisation and *H. pylori* growth in acidic environments^63^. Epiberberine and palmatine might therefore inhibit only extracellular, and not intracellular, urease within 30 min, while coptisine might exert its antibacterial and bactericidal effects against *H. pylori* by inhibiting both intracellular and extracellular urease *in vivo* and *in vitro*. It is possible that the more hydrophobic groups present in the isoquinoline structure of coptisine facilitated its diffusion across *H. pylori* cell membranes, allowing it to inhibit intracellular urease. Additional studies are needed to investigate the mechanisms that might underlie this process.

Urease was first crystalised from jack beans (*Canavalia ensiformis*, JBU); because JBU is the best-characterised urease, it frequently serves as a model urease in studies of inhibitory mechanisms. We therefore compared the inhibitory effects of coptisine on HBU and JBU. Our kinetic study using the Lineweaver–Burk plot revealed that coptisine administration resulted in mixed-type inhibition of HPU, which agreed with previous studies of coptisine-induced inhibition patterns in bacterial collagenase from *Clostridium histolyticum* and in porcine pancreatic elastase^57^^,^^62^. In contrast, coptisine non-competitively inhibited JBU, which was also the case in our earlier reports on Rhizoma Coptidis extract and the alkaloids epiberberine and palmatine^29^^,^^32^^,^^33^. These kinetic mechanism differences might arise from structural differences between HPU and JBU. However, further in-depth investigation of these differences is needed.

Urease is a thiol-rich, nickel-dependent metalloenzyme. Ni^2+^ ions and –SH groups, especially the multiple cysteinyl residues located on the mobile flap that closes over the active site, are essential for the catalytic activity of all ureases^64^. Our results demonstrated that the sulfhydryl reagents DTT, GSH, and L-cys protected against coptisine-induced inhibition of HPU and JBU, and DTT restored urease activity, indicating that coptisine binds to the active site sulfhydryl group to inhibit urease activity. These observations agreed with our previous findings that non-competitive binding and blockage of JBU sulfhydryl groups by Rhizoma Coptidis extract, epiberberine, and palmatine contributed to urease inhibition^29^^,^^32^^,^^33^. Interestingly, the typical competitive urease inhibitors BA and NaF protected against coptisine-induced inhibition of HPU, but had a synergistic effect on coptisine-induced inhibition of JBU. This indicates that the active site Ni^2+^ may be essential for coptisine-induced inhibition of HPU, which was supported by the kinetic analysis, molecular docking simulation, and spectrophotometric studies.

Molecular docking analysis revealed that coptisine formed a hydrogen bond interaction with the NH group of His-221 and a metal-ligand with the active site Ni^2+^ in HPU. In addition, the benzene ring of coptisine might form hydrophobic interactions with Ala-169, Ala-365, Met-366, His-322, Cys-321, and His-323 of HPU (Figure 9(A)). However, coptisine only formed hydrogen bond or hydrophobic interactions with amino acid residues Arg-439, His-519, His-593 Met-637, His-594, and Cys-406 of JBU (Figure 9(B)). Previous studies have shown that these amino acid residues, which are located on the mobile flap that opens and closes during the urease catalytic cycle, are responsible for hydrogen bonding^65–67^. Coptisine tightly anchored the helix-turn-helix motif over the active site cavity through N-H⋯O hydrogen bonding interactions, which prevented the mobile flap from returning to the closed position, thereby inhibiting urease activity. These observations together with the protective effects of active site binding inhibitors in the reactivation assay strongly support the involvement of the active site Ni^2+^ and sulfhydryl groups in coptisine-induced inhibition of HPU while sulfhydryl groups of JBU. The ultraviolet absorption assay revealed that coptisine chelated Ni^2+^ and bound to sulfhydryl groups, which was congruent with a previous study indicating that coptisine alters enzyme activity at least in part by chelating ferrous metal ions^34^. Taken together, our results suggest that coptisine inhibits urease not only by binding to active site sulfhydryl groups, but also via a specific interaction with the Ni^2+^ ion.

Because the catalytic activity of urease requires the presence of Ni^2+^ ions in the active site, *H. pylori* requires efficient acquisition of Ni^2+^ ions to survive^68^^,^^69^. *In vivo*, urease is synthesised as an inactive apoenzyme, and maturation of urease involves delivery of nickel to the metallo-active centre, which is highly dependent on the cooperation of at least four accessory proteins (UreE, UreG, UreF, and UreH)^23^. UreF and UreH form a complex that recruits UreG to the pre-activation complex. Formation of the UreG/UreF/UreH complex is a critical step in urease maturation and formation of the nickel-charged UreG dimer, which reverts to its monomeric form and releases nickel into the urease active site upon GTP hydrolysis^26^. The UreG protein contains a highly conserved nickel-binding motif that is essential for nickel delivery and is required to complete biosynthesis of nickel enzymes^25^^,^^70^. UreG couples GTP hydrolysis to urease activation, and it is thought to catalyse the formation of carboxyphosphate, which results in carbamylation of the metal-binding lysine in the urease active site^15^^,^^24^. In this study, coptisine inhibited activity of the prototypical urease accessory protein UreG (Figure 10) and suppressed nickel-charged UreG dimer formation (Figure 12), without influence on GTP (Figure 11). Furthermore, coptisine dissociated nickel from the UreG dimer (Figure 13), which might be associated with its ability to chelate nickel as observed in the spectrophotometric study. Together, these results suggest that coptisine interferes with the assembly of the pre-activation complex and delivery of Ni^2+^ ions during HPU maturation.

Previous reports have suggested that coptisine promotes gastric mucosal protection more effectively than cimetidine or sucralfate^40^^,^^71^. Acute and sub-chronic toxicity assays indicate that coptisine has a more favourable safety profile than its congener berberine, an effective agent commonly used for the treatment of gastrointestinal disorders in China^72^^,^^73^. At present, the first-line monotherapies for *H. pylori* infection typically have either direct anti-*H. pylori* effects (e.g. antibiotics) or gastroprotective properties (e.g. proton-pump inhibitor). However, none of the standard therapies demonstrate anti-*H. pylori* effects together with urease inhibition and anti-ulcer activity. Because of its anti-*H. pylori*, anti-HPU, and gastroprotective properties, coptisine may be a safe and effective therapeutic agent for the treatment of *H. pylori* infection.

## Conclusion

In this study, we found that coptisine effectively inhibited *H. pylori* growth and urease activity at least in part by interacting with the nickel metallocentre and sulfhydryl groups of the urease active site. Coptisine also inhibited assembly of the pre-activation complex during HPU maturation by interacting with both the prototypical urease accessory protein UreG and nickel ions, potentially contributing to its anti-*H. pylori* effect. These findings suggest that coptisine might be a promising novel candidate *H. pylori* and urease inhibitor, and that the traditional Chinese medicine Rhizoma Coptidis might be effective for treating *H. pylori*-related gastrointestinal diseases.
